# Nécrose corticale laminaire compliquant une piqure de scorpion

**DOI:** 10.11604/pamj.2013.14.124.2274

**Published:** 2013-03-30

**Authors:** Adnane Mohamed Berdai, Mustapha Harandou

**Affiliations:** 1 Service de réanimation Mère et enfant, centre hospitalier Hassan II, Fès, Maroc

**Keywords:** Nécrose corticale, piqure de scorpion, convulsions, coma, cortical necrosis, Scorpion sting, convulsions, coma

## Images en médicine

La nécrose corticale laminaire correspond à une ischémie neuronale associée à une réaction gliale et un dépôt laminaire de macrophages riches en lipides. Elle survient à la suite d'une hypoxie cérébrale, avec atteinte des couches profondes du cortex. La substance grise, plus vulnérable que la substance blanche, peut être atteinte de manière isolée, définissant une nécrose neuronale sélective. Radiologiquement, seule l'imagerie par résonance magnétique (IRM) permet de le détecter et de suivre son évolution, elle correspond à un hypersignal linéaire spontané en pondération T1 de la corticale, d'origine non hémorragique. Elle peut s'observer à la suite d'une hypotension artérielle prolongée, d'une hypoglycémie, d'un état de mal épileptique, d'arrêt cardiaque et respiratoire. Nous rapportons le cas d'un enfant de 20 mois, victime d'une piqure de scorpion au niveau de la cuisse, entrainant initialement des signes inflammatoires locaux, des vomissements, et un priapisme. Rapidement, le tableau clinique s'est aggravé par l'installation d'une détresse respiratoire et des troubles de conscience. L'examen clinique montrait un état de choc et un coma à 7 sur l’échelle de Glascow. L'enfant était intubé et ventilé, il a reçu l'adrénaline en perfusion continue. L'imagerie par résonance magnétique a montré une nécrose laminaire corticale. L'extubation était réalisée 15 jours après, L’évolution est marqué par l'installation de crises convulsives partielles, l'examen neurologique montrait une absence de communication et une tetraparésie spastique.

**Figure 1:**
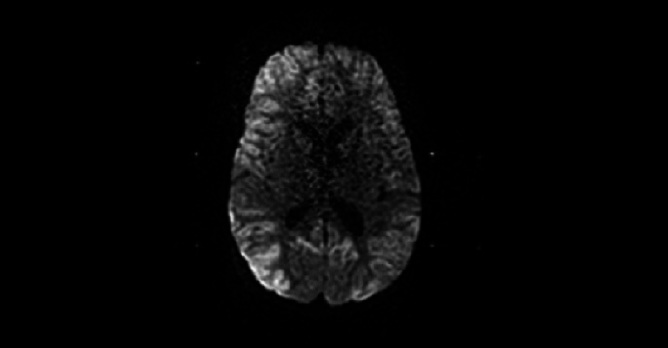
Coupe axiale d'une séquence IRM de diffusion montrant une nécrose laminaire corticale étendue et prédominant au niveau de l'hémisphère droit

